# Age Associated Changes in Transcription of *Adiponectin*, *AdipoR1* and *AdipoR2* Genes in Pancreas of Rats

**DOI:** 10.22074/cellj.2020.6921

**Published:** 2020-09-08

**Authors:** Marziyeh Feyzi, Mohammad Reza Tabandeh, Mehrdad Shariati, Edalatmanesh Mohammad Amin

**Affiliations:** 1. Department of Biology, Fars Science and Research Branch, Islamic Azad University, Fars, Iran; 2. Department of Biology, Shiraz Branch, Islamic Azad University, Shiraz, Iran; 3. Department of Basic Sciences, Division of Biochemistry and Molecular Biology, Faculty of Veterinary Medicine, Shahid Chamran University of Ahvaz, Ahvaz, Iran; 4. Stem Cells and Transgenic Technology Research Center, Shahid Chamran University of Ahvaz, Ahvaz, Iran; 5. Department of Biology, Kazerun Branch, Islamic Azad University, Kazerun, Iran; 6. Department of Biology, College of Sciences, Shiraz Branch, Islamic Azad University, Shiraz, Iran

**Keywords:** *Adiponectin*, *Adiponectin* Receptors, Aging, Pancreas

## Abstract

**Objective:**

Adiponectin has a crucial role in the function, proliferation and viability of β-cell via action of two receptors: *AdipoR1* and *AdipoR2*. Nevertheless, age related change of Adiponectin system genes in pancreas is unclear or controversial. This study sought to investigate the effects of aging process on serum Adiponectin levels, *Adiponectin* and its receptor expression in the rat pancreas.

**Materials and Methods:**

In this experimental study, insulin resistance markers including serum insulin and glucose concentrations, homeostatic model assessment of insulin resistance (HOMA-IR), oral glucose tolerance test (OGTT), glucose induced insulin secretion (GIIS), serum Adiponectin levels, pancreatic expression of Adiponectin and its receptors were studied in male Sprague-Dawley rats at the age of 2, 5, 10, 18, 52 and 72 weeks of age.

**Results:**

We found that aging triggered signs of insulin resistance characteristics in rats at 72 age weeks including marked insulin reduction, hyperglycemia and increased HOMA-IR. Circulating Adiponectin as well as pancreatic expression of *Adiponectin* and AdipoR1 was gradually decreased with age, while the opposite expression pattern of *AdipoR2* was observed in the old rats.

**Conclusion:**

Because Adiponectin and Adiponectin signaling have crucial role in β-cell function and viability, we concluded that reduction of Adiponectin signaling may be involved in aging induced β-cell dysfunction. As a result, manipulation of Adiponectin signaling may be a beneficial approach for improvement of β-cell function in the old people.

## Introduction

Aging is an important risk factor for metabolic disorders, including obesity, impaired glucose tolerance and type 2 diabetes (T2D). T2D has been estimated to be increased monotonically with age, both in animal and human (1, 2). Age associated glucose intolerance, insulin resistance and T2D may result other age related diseases such as cancer, stroke, cardiovascular diseases, Parkinson’s disease and Alzheimer’s disease (3). The pathophysiologic mechanisms underlying age-induced glucose intolerance remain incompletely understood. Because insulin is the main regulator of glucose homeostasis, peripheral insulin resistance, impaired insulin secretion from β-cells and unusual insulin clearance are considered as the major age-related complications in old rodents as well as humans (4). Pancreatic β-cell mass is another factor affecting development of insulin resistance in old animals and human. Proliferation, apoptosis of β-cells and islet neogenesis are three major factors that tightly regulate β-cell mass (5). It has been shown that age correlates with decreased proliferative activity and enhanced sensitivity to glucose-induced β-cell apoptosis (6). Recent finding has demonstrated that in old Wistar rats increasing glucose concentration induced a higher level of cell death and lower level of β-cell proliferation in relation with those in young animals (7).

New findings suggest the close relationship between adipose tissue dysfunction and endocrine pancreatic health. Adipose tissue is now recognized to be an important endocrine organ that secretes biologically active compounds, known as adipokines (8, 9). Despite the ever-expanding list of adipokines, which now accounts for over 300 secretory products, few have been studied on their roles in β-cell function (10).

Adiponectin, as a 30 kDa secretory protein, was one of the earlier adipokines identified in rodents and human (11, 12). Adiponectin circulate primarily as a multimeric (trimeric, hexameric and high molecular weight) polypeptide and is locally proteolytically cleaved to a globular (trimeric) form in which the collagen-like amino-terminal domain is released (12). Adiponectin has antidiabetic properties and its circulating concentrations are reduced in patients with visceral obesity, insulin resistance and T2D (9, 13, 14). Adiponectin performs its physiological effects mainly via AdipoR1 and AdipoR2 receptors. Scatcherd plot analysis revealed that AdipoR1 is a receptor for globular Adiponectin, whereas AdipoR2 is a receptor for full-length Adiponectin. *AdipoR1* is abundantly expressed in muscle, while *AdipoR2* is predominantly expressed in liver (13, 14). Adiponectin -AdipoRs interaction results in activation of different signaling pathways such as AMPK, peroxisome proliferator-activated receptors (PPARs) and p38 MAPK (15).

Recent findings have shown that *Adiponectin* and its two receptors are expressed in β-cells and they have substantial roles in viability and insulin secretion potency of β-cells (15-17). Both *AdipoR1* and *AdipoR2* are expressed in rodent pancreatic beta-cells, while the levels of *AdipoR1* mRNA being expressed at a higher level than *AdipoR2* (16, 17). It has been found that Adiponectin can reverse high glucose induced β-cell impairment and apoptosis in INS-1 clonal rat cells (18). Both globular and truncated Adiponectin (gADN and ADN15-36) stimulate expression of the genes related to function of β-cell including insulin and pancreatic and duodenal homeobox 1 (*PDX1*) gene (mRNA) and they increase viability of β-cells (19). *Adiponectin* gene overexpression or ablation in mice has demonstrated that it can protect β-cell against caspase-8-mediated apoptosis (20).

It is clear that Adiponectin have substantial effects on both function and survival of β-cells. This raises the question of whether *Adiponectin* and its two receptors are changed in aging process of pancreatic beta cells and whether this in turn contributes to the age-related change in insulin sensitivity and glucose stimulated insulin secretion (GSIS),* in vivo*. Therefore, the aim of current study was to define relative expression of *Adiponectin, AdipoR1* and *AdipoR2* in the pancreas and their association with insulin resistance markers in aging process of normal rats.

## Materials and Methods

### Experimental animals

In this experimental study, male Sprague-Dawley rats at different ages were obtained from animal house of faculty of veterinary medicine, Shahid Chamran university of Ahavz (Iran). All rats were housed four per cage in the standard polycarbonate cages with hardwood chip bedding in the Clean Animal Room and they were allowed free access to food and water. They were housed at 22-24˚C and relative humidity of 60% in 12 hours light/dark cycles, with regular ventilation. All experimental protocols were approved by the Ethics Committee of Shahid Chamran University of Ahvaz for animal and human experiments. All the recruited animals were cared according to the guideline for the care and use of laboratory animals by the national academy of sciences (National Institutes of Health publication No. 86-23).

### Sampling

Animals were sacrificed at different ages including; 2 (immature group, n=10), 5 (puberty group, n=10), 10 (puberty group, n=10), 18 (young adult group, n=10), 52 (aged group, n=10) and 72 (aged group, n=10) weeks of age. These numbers were based on natural development of pancreas and life cycle curve of rats, as described previously (21). Half of animals in each age group (n=5) were fasted for 12 hours before sampling and scarified by decapitation under anesthesia using combination of ketamine and xylazine (100 mg/kg of ketamine and 10 mg/kg of xylazine). Blood sample was collected and serum was harvested following centrifugation for 5 minutes at 5000 × g and they were next stored at -20˚C until analysis for hormones and metabolites. Pancreas were removed and kept at -80°C until use. The weight of animals was recorded before scarification.

### Oral glucose tolerance test

Oral glucose tolerance test (OGTT) was performed in half of the animals presented in each age group (n=5). After 12 hours fasting, a 20% glucose solution (2 g/kg body weight) was administered to the rats via a polyethylene gastric tube. Blood glucose was measured by tail prick 15, 30, 90 and 120 minutes using hand-held glucometer (EasyGluco, China) (22). At the end of experiment (120 minutes after glucose administration) blood samples were obtained by heart puncture and serum was separated for determination of insulin concentration. Serum was harvested following the centrifugation for 5 minutes at 5000 × g and it was stored at -20˚C until analysis for hormones and metabolites.

### Homeostatic model assessment of insulin resistance estimation

The homeostasis model assessment of basal insulin resistance (HOMA-IR) was calculated based on fasting concentrations of plasma glucose (mmol/l) and plasma insulin (μU/ml) as described previously. Lower HOMAIR values indicated greater insulin sensitivity, whereas higher HOMA-IR values indicated lower insulin sensitivity (insulin resistance) (23).

### Biochemical assay

Adiponectin concentration was measured in serum by using rat ELISA kit (EastBiopharm Co Ltd, China). The intra- and inter-assay coefficients of variation were 3.6 and 7.4%, respectively. Insulin concentration in serum was determined with a commercially available ELISA kit (Mercodia, Sweden). The intra- and inter-assay coefficients of variation were 6.1 and 8.5%, respectively. Serum glucose concentration was determined using a commercial kit through enzymatic colorimetry assay (Pars Azmoon Co, Iran).

### RNA extraction and cDNA synthesis

Total RNA was isolated from approximately 50 mg pancreas using RNX^plus^ Kit (Sinaclon Inc, Iran) according to manufacturer’s protocol. The yield of extracted RNA was determined spectrophotometrically by measuring the optical density at 260 nm using Eppendorf μCuvette G1.0 microvolume measuring cell (Eppendorf, Germany). The purity and quality of extracted RNA were evaluated using measurement of optical density ratio at 260/280 nm. RNA samples with a ratio more than 1.8 were used for quantitative reverse transcription polymerase chain reaction (qRT-PCR) experiments. For each sample, 0.5 μg of total RNA was reverse transcribed by YTA cDNA synthesis kit using random primers as described by the manufacturer (Yektatajhiz, Iran).

### Quantitative reverse trasncription polymerase chain reaction analysis

qRT-PCR was carried out on a Lightcycler Detection System (Roche, USA) using qPCR^TM^ Green Master Kit for SYBR Green I^®^ according to the manufacturer’s recommendation (Yektatajhiz, Iran). Reactions were carried out in a 12.5 μl total volume containing 6.25 μl qPCR^TM^ Green Master Kit for SYBR Green I^®^ (Yektatajhiz, Iran), 0.25 μl of each primer (200 nM), 3 μl cDNA (100 ng) and 2.25 μl nuclease-free water. The following specific primers were used for:

*Adiponectin*-

F: 5´-AATCCTGCCCAGTCATGAAG-3´

R: 5´-CATCTCCTGGGTCACCCTTA-3´, (GeneBank Accession No: NM_144744),

*Adiponectin Receptor 1 (AdipoR1)*-

F: 5´-CTTCTACTGCTCCCCACGGC-3´

R: 5´-TCCCAGGAACACTCCTGCTC-3´, (GeneBank Accession No: XM_006249852.3),

*Adiponectin Receptor 2 (AdipoR2)*-

F: 5´-CCACACAACACAAGAATCCG-3´

R:5´-CCCTTCTTCTTGGGAGAATGG-3´, (GeneBank Accession No: 006237183.2)

GAPDH-

F: 5´-AGTTCAACGGCACAGTCAAG-3´

R: 5´-TACTCAGCACCAGCATCACC-3´. (Takapouzist, Iran).

PCR protocol was consisted of 5 minutes denaturation at 94°C followed by 45 cycles of 94˚C for 15 seconds and 60˚C for 30 seconds. Two separate reactions without cDNA or with RNA were performed in parallel as controls. Gene expression level of each sample was standardized to the house-keeping gene, *GAPDH* (GenBank: NM-017008) using the ∆∆Ct method. The relative gene expression levels were determined using the comparative threshold cycle (2^-∆∆Ct^) method and Lightcycler 96^®^ software. Primer amplification efficiency of the individual genes was performed as previously described (24). All qRT PCR analysis was performed according to The Minimum Information for Publication of Quantitative Real-Time PCR Experiments (MIQE) guideline (25).

### Statistical analysis

All data were expressed as means ± standard error of mean (SEM) and analyzed with SPSS software, version 18.0 (IBM SPSS Inc, USA). One way analysis of variance (ANOVA) and Tukey post hoc test were applied for multiple comparisons between groups. A value of P<0.05 was considered statistically significant.

## Results

### Age related changes of weight, insulin and glucose concentrations

**Fig.1:**
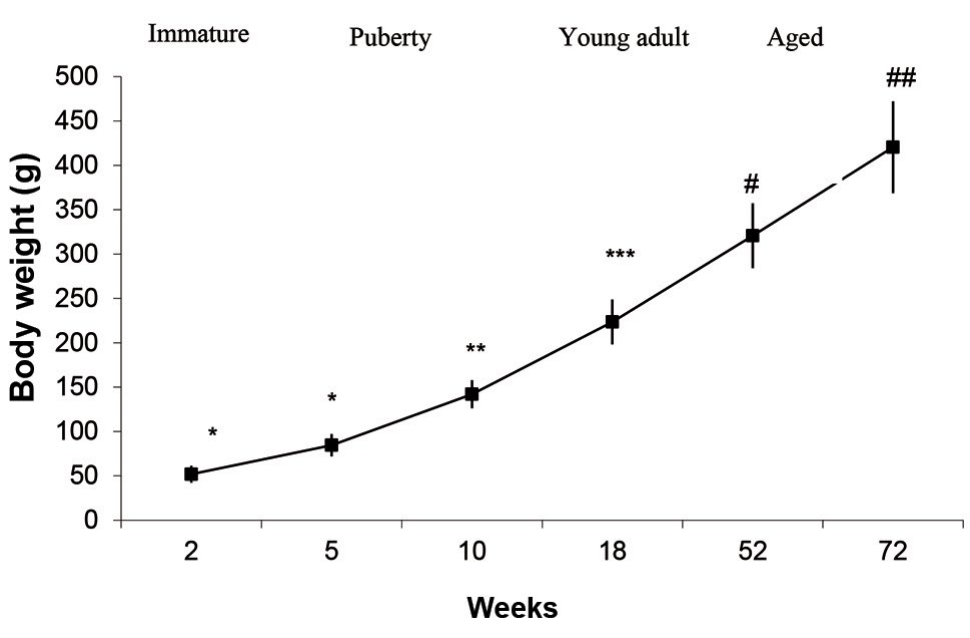
Age-related changes in the body weight of immature (2 weeks old), puberty (5 and 10 weeks old), young adult (18 weeks old) and aged (52 and 72 years old) healthy rats. Data represent means ± SEM for 5 animals in each age group. Comparisons between the groups labeled with different marks were statistically significant (P<0.05).

**Fig.2:**
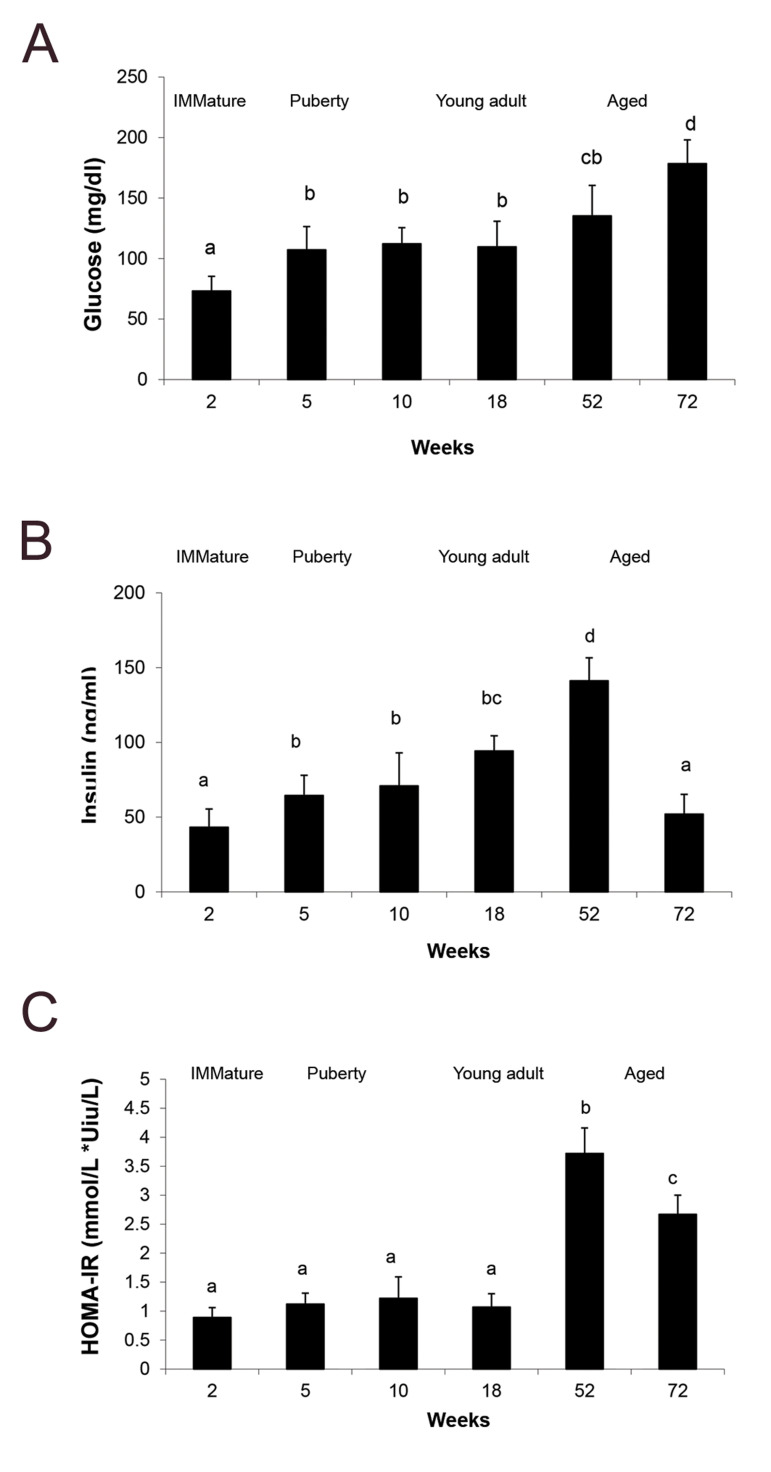
The mean ± SEM for biochemical parameters related to insulin sensivity. **A. **Fasting serum glucose, **B.** Insulin, and **C.** Homeostatic model assessment of insulin resistance (HOMA-IR) values that were examined in immature (2 weeks old), puberty (5 and 10 weeks old), young adult (18 weeks old), and aged (52 and 72 years old) healthy rats. Data was collected from five animals in each age group. Comparisons between the groups labeled with different letters were statistically significant (P<0.05).

As shown in Figure 1, body weight was increased steadily between 5 and 72 weeks of age. Thus, it reached the maximal level at 72 weeks of age (P=0.024). As demonstrated in Figure 2A, glucose concentration had the minimum value in the rats with 2 weeks of age (P<0.05), while it increased in an age dependent manner. Glucose concentration showed constant level in the 5, 10 and 18 weeks old rats and it reached the maximum level in advanced age groups (52-72 weeks of age, P<0.05, Fig .2A). Additionally, changes in serum insulin levels of the rats through aging process have been shown in Figure 2B. Serum insulin concentration showed constant level in the rats with 5, 10 and 18 weeks of age, while it reached the maximum level in 52 weeks age group (P<0.05, Fig .2B). The median insulin level was decreased with advancing age in 72 weeks old group (P<0.05, Fig .2B) .Our results showed no change in *HOMA-IR* of different age groups (2-18 weeks of age, Fig .2C), while it was increased in an age dependent manner in the 52 and 72 weeks old rats (P<0.05, Fig .2C). The highest level of HOMA-IR was determined in the 52 weeks old rats (P<0.05, Fig .2C).

### Effect of aging on oral glucose tolerance test and glucose stimulated insulin secretion

**Fig.3:**
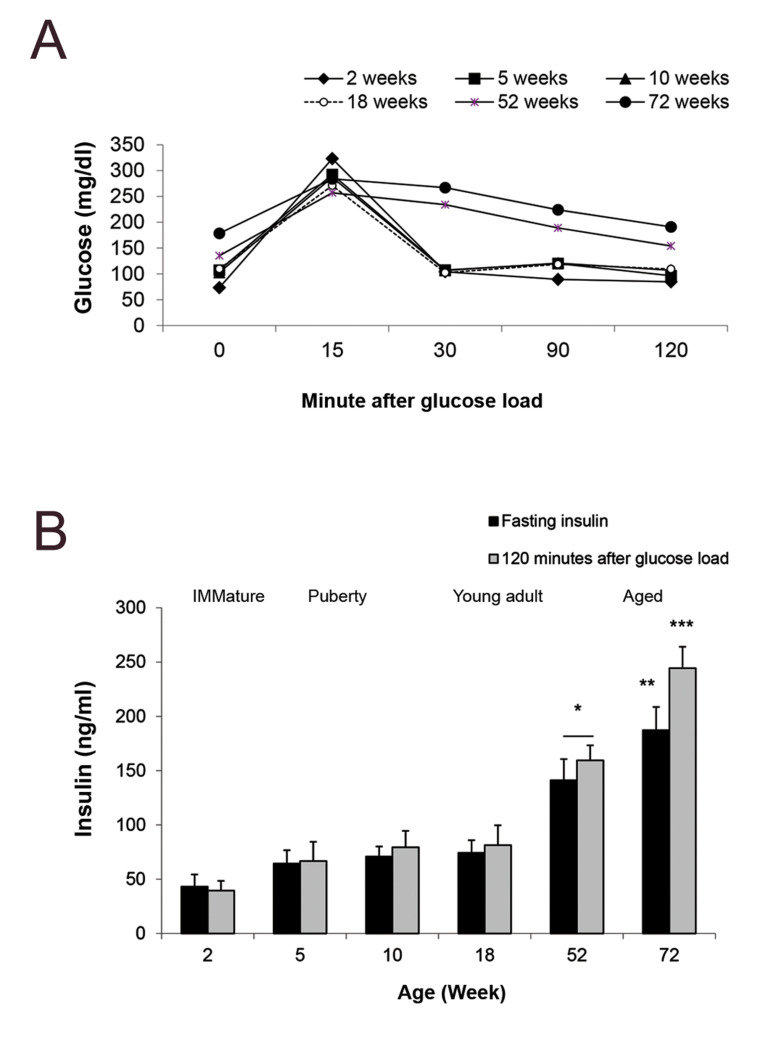
Results of oral glucose tolerance test (OGTT) and glucose stimulated insulin secretion test (GSIS) during aging of rats. **A.** Blood glucose values during the OGTT. Data was collected before and 15, 30, 60, 120 minutes after oral administration of 20% glucose solution in healthy rats with different ages, between 2-72 weeks old. **B.** Blood insulin concentration during the glucose stimulated insulin secretion test (GSIS). Insulin concentration was examined before and 120 minutes after oral administration of 20% glucose solution in healthy rats with different ages, between 2-72 weeks old. Data was collected from five animals in each age group. Comparisons between the groups labeled with different marks were statistically significant (P<0.05).

Figure 3A shows levels of the blood glucose before and after (15, 30, 90, 120 minutes) glucose load during oral glucose tolerance test. As expected, oral glucose administration resulted in an immediate increase in the blood glucose level, which peaked at 15 minutes and then gradually returned to baseline over the following 30 minutes, in young animals. It was found that glucose concentration returned to normal value 30 minutes after OGTT in rats younger than 18 weeks of age, while the 52 and 72 weeks old age groups were increased glucose concentration at the end of OGTT (Fig .3A).

Results verified that 120 minutes after glucose ingestion, blood insulin concentration of the aged groups (52 and 72 weeks old) tended to be higher than those of the younger rats. In the animals with 2, 5, 10 and 18 weeks of age, insulin level returned to fasting level, 2 hours after glucose load. While in the 52 and 72 weeks old rats, insulin concentration was remained high until the end of experiment (P<0.05, Fig .3B).

### Alteration of serum Adiponectin level during aging

**Fig.4:**
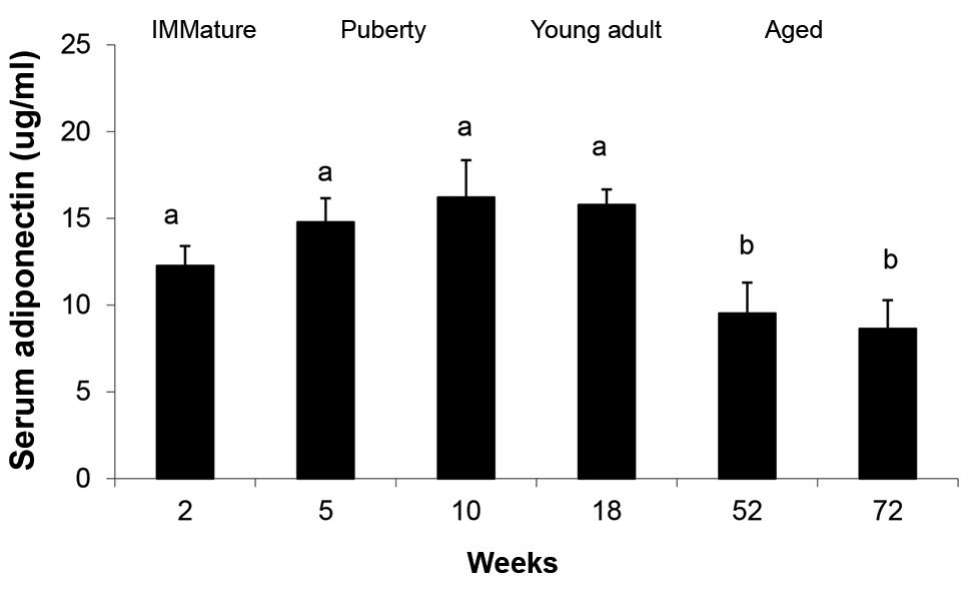
Age-related changes in serum Adiponectin concentration of immature (2 weeks old), puberty (5 and 10 weeks old), young adult (18 weeks old), and aged (52 and 72 years old) healthy rats. Data represent means ± SEM for five animals in each age group. Comparisons between the groups labeled with different marks were statistically significant (P<0.05).

Our results showed no significant difference in serum Adiponectin concentration detected in the rats with age of 2, 5, 10 and 18 weeks (P>0.05), while it was gradually decreased in old rats with 52-72 weeks of age (P<0.05, Fig .4).

### Age related changes in expression of *Adiponectin* and its two receptors in pancrease

**Fig.5:**
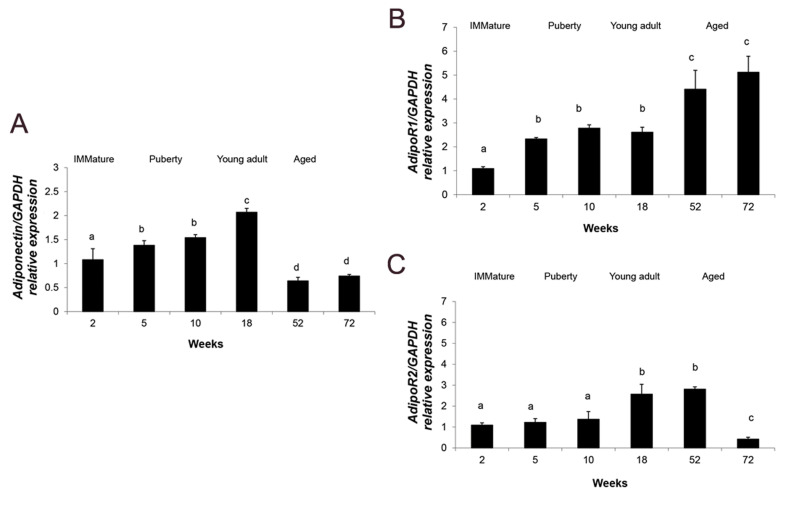
Relative expedition of *Adiponectin* and it’s to receptors in pancreas of rats at different ages. mRNA levels of **A.** *Adiponectin*, **B.** *AdipoRI* and **C. ***AdipoRII* in pancrease of immature (2 weeks old), puberty (5 and 10 weeks old), young adult (18 weeks old) and aged (52 and 72 years old) healthy rats. Data represent means ± SEM for five animals in each age group. Comparisons between the groups labeled with different marks were statistically significant (P<0.05).

An age dependent increase was observed in pancreatic expression of *Adiponectin* in the rats with ages of 2, 5, 10 and 18 weeks (P<0.05, Fig .5A). *Adiponectin* mRNA levels were decreased in the aged rat groups (72 weeks old group) exhibiting the lowest transcription level (P<0.05, Fig .5A). As illustrated in Figure 5B, expression of *AdipoR1* gene in rat aged between 5 and 10 weeks was constant, while it showed significant upregulation at 18 and 52 weeks of age (P<0.05). *AdipoR1* transcription was reduced to the minimum level in the 72 weeks old group (P<0.05). Figure 5C shows the expression levels of *AdipoR2* in pancreas of rats with different ages. It was found that the rats between 2 and 10 weeks of age had similar *AdipoR2* transcription level. *AdipoR2* mRNA level was significantly higher in the 18 and 52 weeks old rats compared to younger animals (P<0.05). *AdipoR2* expression was significantly reduced to the minimum level in the 72 weeks old rats (P<0.05, Fig .5C).

## Discussion

Normal aging is usually associated with progressive β-ell dysfunction that may be responsible for serious disturbances of physiological homeostasis, particularly, glucose tolerance (2, 4, 5). Recently, there has been increasing evidences that Adiponectin, one of the most abundant circulating adipokines, is involved in the regulation of pancreatic β-cell function (10, 14-16). Although expression and function of Adiponectin have been studied in β-cells, little data are available regarding the effect of aging on the expression of *Adiponectin* system genes in pancreas among the aging process. The current study analyzed pancreatic expression of *Adiponectin*, its two receptors and several metabolic markers related to glucose tolerance in normal aging process of rat.

In agreement with the previous studies in both rodents and humans, our results showed that ageing can change anthropometric characteristics and insulin resistance features, such as increasing body weight, serum glucose concentration and HOMA-IR (22, 23). The results of the OGTT and GSIS at 2, 5, 10, 18, 52 and 72 weeks of age of rats demonstrated that glucose tolerance and insulin sensitivity were affected in old animals. Our results showed that, beginning of aging in 52 weeks old animals was associated with marked elevation of serum insulin and glucose levels, while in the advanced age group (72 weeks old animals) insulin level was decreased to the minimum level. Increase in serum insulin levels at the beginning of aging was not in accordance with our assumptions. These changes may be described by the recently coined term "glucose allostasis" theory indicating that the slightly higher glucose levels in insulin-resistant states drive the β-cells to produce higher levels of insulin (26). Glucose allostasis may be a compensatory mechanism can regulate normal glucose metabolism during insulin resistance. Moreover, recent researches have shown that hyperinsulinemia along with moderate hyperglycemia are the first events in developing insulin resistance in human and experimental animals. In accordance with our observation, *Marban* and coworkers showed that mice, stably transfected with extra copies of the human insulin gene, elevated basal plasma insulin level despite the normal weight and fasting glucose, but they display elevated postprandial glucose and diminished insulin tolerance test (27). Taken together we concluded that the onset of aging is associated with hyperinsulinemia, moderate hyperglycemia and diminished GGT, while advanced aging is accomplished with reduction of insulin production along with elevated glucose concentration.

Our results showed reduction of serum Adiponectin in the aged rats. In accordance with our findings, Li et al. (28) showed that plasma Adiponectin levels and visceral fat ratio in the 24months old mice were lower compared to the 2 and 6months old mice. Mori et al. (29) has also shown that circulating Adiponectin level were remarkably lower in dogs aged 8-12 years than dogs aged 0-7 years. Reduction of plasma Adiponectin concentration in early phases of obesity and in parallel to the progressive development of insulin resistance has been reported in obese and diabetic rhesus monkey (30). Prospective studies in Pima Indians demonstrated that high concentrations of Adiponectin were protective against the development of T2D (31). Furthermore, circulating plasma Adiponectin levels and expressions of both *AdipoRs* are reduced in the subjects with a family history of T2D (32). Based on the incidence of insulin resistance in aged rats and previous data, it was concluded that circulating Adiponectin level was reduced with age, indicating that Adiponectin probably played an important role in aging-related diseases, such as insulin resistance. To support this hypothesis, Yamauchi et al. (33) showed that administration of Adiponectin can lead to improvement of insulin sensitivity, glucose tolerance and correction obesity-related hyperglycemia.

Our results showed that *Adiponectin* and *AdipoR2* expression were down-regulated in pancreas of old animals with 72 weeks of age, while *AdipoR1* expression was increased in the advanced age. Recent findings have clarified that Adiponectin and Adiponectin signaling is important regulators of β-cell function and defects in the expression/secretion of *Adiponectin* or its receptors in pancreas of the old rats may be a contributory factor to β-cell dysfunction. This could diminish insulin secretion during the aging process. Recently Okamoto et al. (34) has shown that Adiponectin stimulates insulin secretion from pancreatic islets, confirming that down-regulation of *Adiponectin* in pancreas of the old rats may affect insulin expression or secretion among the aging process. Molecular *reason* for observing opposite pattern of expression of *AdipoR1* and *AdipoR2* in pancreas of the aged animals is unknown. *AdipoR1* act as a major receptor of Adiponectin in pancreas, regarding the level of *AdipoR1* mRNA expressed at a higher level than *AdipoR2* in pancreas (16, 17). Increased *AdipoR1* expression in pancreas of the aged rats may act as a compensatory mechanism to restore the *Adiponectin* signaling and sensitize the pancreatic cells to the Adiponectin, when its expression or secretion is reduced. Further investigations are needed to give a better insight into the molecular mechanisms of Adiponectin receptors transcription in the aged animals.

Several researchers shifted the focus of their interest on the effect of aging on biology of islet, with particular attention to the proliferative capacity and apoptosis of β-cells. Adiponectin is a candidate molecule because it is a positive regulator of pancreatic β mass. In line with these observations, findings have shown that Adiponectin activates Erk and Akt in clonal β-cells, increases their proliferative activity and regulates their cell cycle (15). It has been found that in INS-1 clonal β-cells, Adiponectin can protect against palmitate or ceramide induced apoptosis (35). Furthermore *Adiponectin* gene overexpression in mice can attenuate caspase-8 mediated apoptosis in β-cells (36). Recently, transcriptome analysis revealed that Adiponectin can boost β-cells regeneration by improving pancreatic islet lipid metabolism and this antilipotoxic effect are attributed to up-regulation of two key transcription factors, including hepatocyte nuclear factor 4 (HNF4) and peroxisome proliferator activated receptor α (PPARα) (35). These data suggest that Adiponectin can maintain a normoglycaemic environment in pancreatic islet in the face of decreases in insulin sensitivity. This could be performed preservation of the β-cell mass and diminishing *Adiponectin* expression or its signaling pathway. These alterations may have important physiological role in progressing β-cell dysfunction and decreasing insulin secretion in the old animals.

One of the major finding of our study was the increased expression of pancreatic *AdipoR1* in the aged rats, despite up-regulation of *Adiponectin* and *AdipoR1*. It remains unclear whether an increase in *AdipoR1* expression of the pancreas with age is a positive feedback regulation induced by decreased *Adiponectin* expression, or the compensatory mechanism for improvement of Adiponectin action in pancrease, which deserves further investigation.

## Conclusion

Circulating Adiponectin as well as the pancreatic expression of *Adiponectin* and *AdipoR1* reduced with age, which is accompanied by the increased insulin resistance markers in old rats. Because Adiponectin and Adiponectin signaling have crucial role in β-cell function and viability, we concluded that reduction of Adiponectin signaling may be involved in aging induced β-cell dysfunction and related metabolic complications, in old animals or humans. Given the findings of our study direct activation of the *Adiponectin* receptors, via small molecule agonists, may be used in future for modulation of Adiponectin system and improvement of β-cell function in the aged people.

## References

[1] Lee PG, Halter JB (2017). The pathophysiology of hyperglycemia in older adults: clinical considerations. Diabetes Care.

[2] De Tata V (2014). Age-related impairment of pancreatic Beta-cell function: pathophysiological and cellular mechanisms. Front Endocrinol (Lausanne).

[3] Yeap BB (2013). Hormones and health outcomes in aging men. Exp Gerontol.

[4] Gunasekaran U, Gannon M (2011). Type 2 diabetes and the aging pancreatic beta cell. Aging (Albany NY).

[5] Aguayo-Mazzucato C, van Haaren M, Mruk M, Lee TB Jr, Crawford C, Hollister-Lock J (2017). β Cell aging markers have heterogeneous distribution and are induced by insulin resistance. Cell Metab.

[6] Tschen SI, Dhawan S, Gurlo T, Bhushan A (2009). Age-dependent decline in cell proliferation restricts the capacity of cell regeneration in mice. Diabetes.

[7] Gu Z, Du Y, Liu Y, Ma L, Li L, Gong Y (2012). Effect of aging on islet beta-cell function and its mechanisms in Wistar rats. Age (Dordr).

[8] Tabandeh MR, Jafari H, Hosseini SA, Hashemitabar M (2015). Ginsenoside Rb1 stimulates adiponectin signaling in C2C12 muscle cellsthrough up-regulation of AdipoR1 and AdipoR2 proteins. Pharm Biol.

[9] Nazari M, Moghimipour E, Tabandeh MR (2017). Betaine down regulates apelin gene expression in cardiac and adipose tissues of insulin resistant diabetic rats fed by high-calorie diet. Int J Peptide Res Therap.

[10] Dunmore SJ, Brown JE (2013). The role of adipokines in β-cell failure of type 2 diabetes. J Endocrinol.

[11] Wang ZV, Scherer PE (2016). Adiponectin, the past two decades. J Mol Cell Biol.

[12] Scherer PE, Williams S, Fogliano M, Baldini G, Lodish HF (1995). A novel serum protein similar to C1q, produced exclusively in adipocytes. J Biol Chem.

[13] Yamauchi T, Kamon J, Ito Y, Tsuchida A, Yokomizo T, Kita S (2003). Cloning of adiponectin receptors that mediate antidiabetic metabolic effects. Nature.

[14] Ruan H, Dong LQ (2016). Adiponectin signaling and function in insulin target tissues. J Mol Cell Biol.

[15] Wijesekara N, Krishnamurthy M, Bhattacharjee A, Suhail A, Sweeney G, Wheeler MB (2010). Adiponectin-induced ERK and Akt phosphorylation protects against pancreatic beta cell apoptosis and increases insulin gene expression and secretion. J Biol Chem.

[16] Wade TE, Mathur A, Lu D, Swartz-Basile DA, Pitt HA, Zyromski NJ (2009). Adiponectin receptor-1 expression is decreased in the pancreas of obese mice. J Surg Res.

[17] Kharroubi I, Rasschaert J, Dizirik DL, Cnop M (2003). Expression of adiponectin receptors in pancreatic b cells. Biochem Biophy Res Communic.

[18] Lin P, Chen L, Li D, Liu J, Yang N, Sun Y (2009). Adiponectin reduces glucotoxicity-induced apoptosis of INS-1 rat insulin-secreting cells on a microfluidic chip. Tohoku J Exp Med.

[19] Brown JE, Conner AC, Digby JE, Ward KL, Ramanjaneya M, Randeva HS (2010). Regulation of beta-cell viability and gene expression by distinct agonist fragments of adiponectin. Peptides.

[20] Liadis N, Salmena L, Kwan E, Tajmir P, Schroer SA, Radziszewska A (2007). Distinct in vivo roles of caspase-8 in beta-cells in physiological and diabetes models. Diabetes.

[21] Bonner-Weir S, Aguayo-Mazzucato C, Weir GC (2016). Dynamic development of the pancreas from birth to adulthood. Ups J Med Sci.

[22] Ghezzi AC, Cambri LT, Botezelli JD, Ribeiro C, Dalia RA, de Mello MA (2012). Metabolic syndrome markers in wistar rats of different ages. Diabetol Metab Syndr.

[23] Bowe JE, Franklin ZJ, Hauge-Evans AC, King AJ, Persaud SJ, Jones PM (2014). Assessing glucose homeostasis in rodent models. J Endocrin.

[24] Tabandeh MR, Hosseini A, Saeb M, Kafi M, Saeb S (2010). Changes in the gene expression of adiponectin and adiponectin receptors (AdipoR1 and AdipoR2) in ovarian follicular cells of dairy cow at different stages of development. Theriogenology.

[25] Bustin SA, Benes V, Garson JA, Hellemans J, Huggett J, Kubista M (2009). The MIQE guidelines: Minimum information for publication of quantitative real-time PCR experiments. Clin Chem.

[26] Cerf ME (2013). Beta cell dynamics: beta cell replenishment, beta cell compensation and diabetes. Endocrine.

[27] Marban SL, Roth J (1996). Transgenic hyperinsulinemia: a mouse model of insulin resistance and glucose intolerance without obesity.Shafrir E, editor.In: Lessons from animal diabetes VI.

[28] Li B, Nishida M, Kaimoto K, Asakawa A, Chaolu H, Cheng KC (2014). Effects of aging on the plasma levels of nesfatin-1 and adiponectin. Biomed Rep.

[29] Mori N, Kawasumi K, Arai T (2012). Comparison of the plasma insulin and adiponectin concentrations as metabolic markers in clinically healthy dogs with ageing. J Anim Vet Adv.

[30] Hotta K, Funahashi T, Bodkin NL, Ortmeyer HK, Arita Y, Hansen BC (2001). Circulating concentrations of the adipocyte protein adiponectin are decreased in parallel with reduced insulin sensitivity during the progression to type 2 diabetes in rhesus monkeys. Diabetes.

[31] Lindsay RS, Funahashi T, Hanson RL, Matsuzawa Y, Tanaka S, Tataranni PA (2002). Adiponectin and development of type 2 diabetes in the Pima Indian population. Lancet.

[32] Civitarese AE, Jenkinson CP, Richardson D, Bajaj M, Cusi K, Kashyap S (2004). Adiponectin receptors gene expression and insulin sensitivity in non-diabetic Mexican Americans with or without a family history of Type 2 diabetes. Diabetologia.

[33] Yamauchi T, Kamon J, Waki H, Terauchi Y, Kubota N, Hara K (2001). The fat-derived hormone adiponectin reverses insulin resistance associated with both lipoatrophy and obesity. Nat Med.

[34] Okamoto M, Ohara-Imaizumi M, Kubota N, Hashimoto S, Eto K, Kanno T (2008). Adiponectin induces insulin secretion in vitro and in vivo at a low glucose concentration. Diabetologia.

[35] Ye R, Wang M, Wang QA, Scherer PE (2015). Adiponectin-mediated antilipotoxic effects in regenerating pancreatic islets. Endocrinology.

[36] Lin P, Chen L, Li D, Liu J, Yang N, Sun Y (2009). Adiponectin reduces glucotoxicity-induced apoptosis of INS-1 rat insulin-secreting cells on a microfluidic chip. Tohoku J Exp Med.

